# Bone Erosions Detected by Ultrasound Are Prognostic for Clinical Arthritis Development in Patients With ACPA and Musculoskeletal Pain

**DOI:** 10.3389/fmed.2021.653994

**Published:** 2021-03-23

**Authors:** Michael Ziegelasch, Emma Eloff, Hilde B. Hammer, Jan Cedergren, Klara Martinsson, Åsa Reckner, Thomas Skogh, Mattias Magnusson, Alf Kastbom

**Affiliations:** ^1^Department of Rheumatology, Department of Clinical and Experimental Medicine, Linköping University, Linköping, Sweden; ^2^Department of Rheumatology, Diakonhjemmet Hospital, Oslo, Norway

**Keywords:** anti-citrullinated protein antibodies, rheumatoid arthritis, ultrasound, musculoskeletal pain, erosions, clinical arthritis

## Abstract

Anti-citrullinated protein antibodies (ACPA) often precede onset of rheumatoid arthritis (RA) by years, and there is an urgent clinical need for predictors of arthritis development among such at-risk patients. This study assesses the prognostic value of ultrasound for arthritis development among ACPA-positive patients with musculoskeletal pain. We prospectively followed 82 ACPA-positive patients without clinical signs of arthritis at baseline. Ultrasound at baseline assessed synovial hypertrophy, inflammatory activity by power Doppler, and erosions in small joints of hands and feet. We applied Cox regression analyses to examine associations with clinical arthritis development during follow-up (median, 69 months; range, 24–90 months). We also compared the ultrasound findings among the patients to a control group of 100 blood donors without musculoskeletal pain. Clinical arthritis developed in 39/82 patients (48%) after a median of 6 months (range, 1–71 months). One or more ultrasound erosions occurred in 13/82 patients (16%), with none in control subjects (*p* < 0.001). Clinical arthritis development was more common among patients with baseline ultrasound erosions than those without (77 vs. 42%, *p* = 0.032), and remained significant in a multivariable Cox regression analysis that included previously described prognostic factors (HR 3.9, 95% CI 1.6–9.4, *p* = 0.003). Ultrasound-detected tenosynovitis was more frequent among the patients and associated with clinical arthritis development in a univariable analysis (HR 2.5, 95% CI 1.1–5.7, *p* = 0.031), but did not remain statistically significant in multivariable analysis. Thus, bone erosions detected by ultrasound are independent predictors of clinical arthritis development in an ACPA-positive at-risk population.

**Trial Registration:** Regional Ethics Committee in Linköping, Sweden, Dnr M220-09. Registered 16 December 2009, https://etikprovningsmyndigheten.se/.

## Background

Autoimmune features, such as the presence of circulating rheumatoid factor (RF) and/or anti-citrullinated protein antibodies (ACPA), typically precede the onset of clinically manifest rheumatoid arthritis (RA) (1, 2), as defined by the 1987 American College of Rheumatology (ACR87) or the 2010 ACR/EULAR classification criteria (3, 4). Neither of these RA classification criteria is applicable to patients who are suffering from musculoskeletal (MSK) pain in the absence of clinical synovitis. However, given the benefits of modern early immunomodulatory therapies for RA (5) and the high diagnostic specificity of ACPA (6), patients within this category may benefit from anti-rheumatic drug therapy prior to fulfilling the classification criteria for RA. Nonetheless, considering the substantial risk of over-treatment with potent agents of immunomodulation in this clinical setting, there is a pressing need for predictors of disease development and progression. Ultrasound, which is an imaging modality that allows the detection of subclinical inflammation in musculoskeletal structures (7), could be valuable in identifying patients who could benefit from very early treatment.

Gray scale (GS) ultrasound visualizes thickening of the synovial membranes (synovial hypertrophy; SH) in joints and tendons, effusions, and structural bone changes, such as erosions (8). The addition of power Doppler (PD) to GS ultrasound findings allows for the detection of hyperemia, which is a sign of active inflammation (9). The use of MSK ultrasound to detect ongoing inflammation and, thereby, predict clinical arthritis development has shown potential in different at-risk populations (10–13), in particular regarding PD (14). However, there are divergent results regarding both the value of each ultrasound feature and whether or not they are predictive at the patient level (14, 15). Also, ultrasound findings of arthritis may occur among non-arthritic controls, although the frequency and magnitude need to be further elucidated. Previous smaller studies have suggested that SH, particularly in the toes, may occur frequently in control populations without clinical arthritis (12, 14, 16).

In experienced hands, ultrasound appears to be more sensitive than conventional radiography for the detection of minimal structural changes located at bone surfaces, at least in certain anatomic sites such as the MCP II and the MCP V (17, 18). However, the prognostic value of ultrasound-detected erosions has been much less studied than SH and PD. One previous study reported a significant association between baseline ultrasound-detected erosions and subsequent development of arthritis, albeit without adjusting for possible confounders (14).

Ultrasound is increasingly used in clinical practice. In patients with RA-related autoantibodies and arthralgia, but no clinical arthritis, it is used for risk stratification and occasionally used for deciding on the initiation of disease-modifying anti-rheumatic drugs (DMARDs). However, a recent literature review concluded that the available evidence remains limited to moderate regarding the prognostic value of SH and PD, and insufficient concerning tenosynovitis and erosions (13).

Therefore, to fill these knowledge gaps, we compared the ultrasound findings of ACPA-positive patients with MSK pain but no clinical arthritis to the findings of healthy controls and investigated the prognostic value of ultrasound findings for subsequent clinical arthritis development.

## Methods

### Patients and Control Subjects

**Figure 1:**
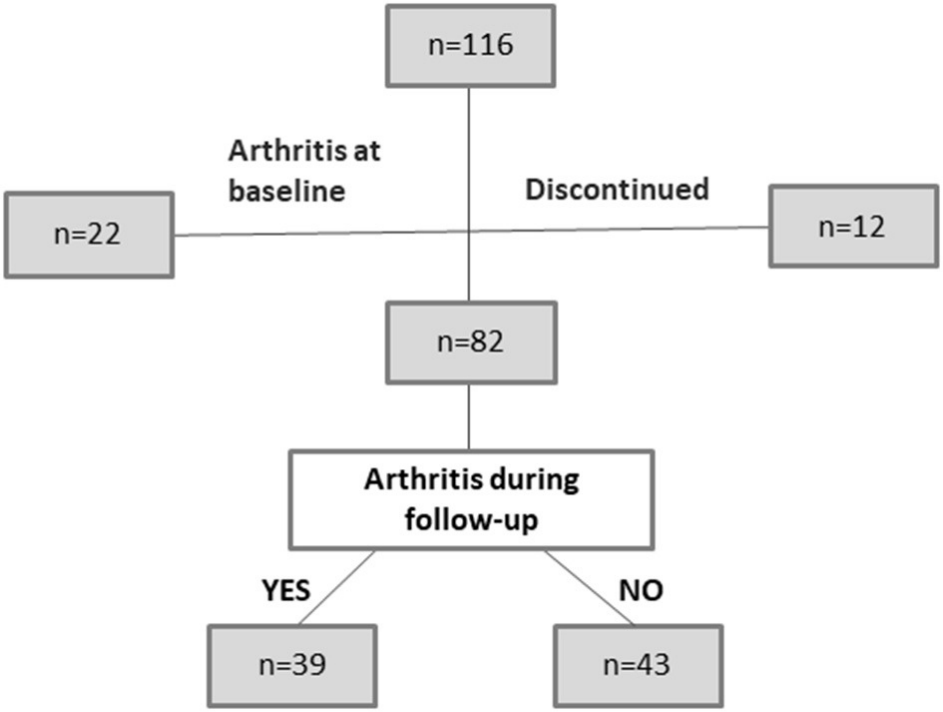
Distribution of patients during the study.

We set up a prospective observational study, designated “TIRx” (Swedish acronym for “X-tra early rheumatology follow-up”), which enrolled 116 patients in the period of 2010–2013 at the University Hospital in Linköping, Sweden. The patients were referred from primary care centers within the Östergötland County in southeast Sweden to the rheumatology clinic, based on ACPA-positivity and any kind and duration of MSK symptom. Screening, enrolment, and follow-up were performed by four experienced rheumatologists (AK, JC, TS, and ÅR). In this study we included patients with MSK pain of any sort and duration and a positive anti-cyclic citrullinated peptide (anti-CCP) antibody test in clinical routine practice. The exclusion criteria were: fulfillment of the ACR1987 criteria (3); oral or intraarticular corticosteroid therapy within 6 weeks prior to screening; previous diagnosis of inflammatory rheumatic disease; and age <18 years. Twelve patients (10%) discontinued and 22 (19%) had clinical arthritis at baseline. Thus, 82 ACPA-positive at-risk patients were available for further analysis (Figure 1). The baseline characteristics of the study subjects are shown in Table 1. Follow-up visits were scheduled at months 3, 12, 24, and 36, and thereafter every other year. Patients were instructed to contact the clinic without delay in case of increased symptoms between scheduled visits. At each visit, we obtained a 28-joint disease activity score (DAS28) (19) and conducted a clinical examination of symptomatic joint(s) not included in the 28-joint status. Pharmacotherapy and non-pharmacologic interventions were instituted as suggested by the physician and with the patient's acceptance. Development of arthritis was defined by clinical examination conducted by an experienced rheumatologist. Follow-up was until September 1st 2017, resulting in a median follow-up time of 69 months [range, 24–90 months, interquartile range (IQR) 57–77] for those patients who did not develop arthritis.

**Table 1:** Baseline characteristics of study participants.

**Characteristic**		**Patients (*N* = 82)**	**Controls (*n* = 100)**
Age, years		52 (14)	52 (14)
Gender, females		66 (81%)	50 (50%)
Symptom duration	0–6 months	15 (18%)	
	6–12 months	37 (45%)	
	>18 months	30 (37%)	
ACPA-level	Low (<3 × cutoff)	32 (39%)	
	High (≥3 × cutoff)	50 (61%)	
RF	Negative	58 (71%)	
	Positive	24 (29%)	
CRP, mg/L		6 (6.0)	
ESR, mm/h		12 (9.5)	
DAS28		2.5 (1.1)	
Smoking	Non-smoker	43 (52%)	
	Ex-smoker	26 (32%)	
	Current smoker	13 (16%)	

*ACPA, Anti-citrullinated protein antibodies; CRP, C-reactive protein; DAS, disease activity score; ESR, erythrocyte sedimentation rate; HAQ, health assessment questionnaire; RF, rheumatoid factor; SD Standard deviation. Values are mean (SD) unless otherwise indicated*.

As controls, we recruited 100 blood donors without MSK pain (Table 1) from the Department of Transfusion Medicine at Linköping University Hospital. This control group did not have arthralgia and was selected so as to have a similar mean age as the TIRx patient group, and underwent ultrasound examination once according to the procedure described below. One out of the 100 healthy controls tested positive for ACPA, which is an expected number given the specificity of the test.

### Ethics Approval and Consent to Participate

The study was conducted in accordance with the principles of the Helsinki Declaration. The study protocol was approved by the Regional Ethics Committee in Linköping, Sweden (DnR 220-09 and 2015/236-32), and all participants gave written informed consent to participate.

### Ultrasound Examinations

All ultrasound examinations were performed by an experienced rheumatologist (MZ). The ProFocus system from BK Medical (BK Global Headquarters, Peabody, MA) with a linear scanner at 6–15 MHz was used. Synovial hypertrophy and bone erosions were assessed with identical GS settings for all participants (B-mode frequency, 12 MHz; B-mode gain, 25 dB), while inflammatory activity was assessed by power Doppler (frequency, 7.5 MHz; Doppler gain 44dB; pulse repetition frequency, 0.8 kHz; and the lowest possible wall filter to avoid artifacts). The protocol included dorsal assessments of the following 36 joints: bilateral radiocarpal, intercarpal, distal radioulnar, metacarpophalangeal (MCP) joints 1–5, interphalangeal (IP) thumb joints, proximal interphalangeal (PIP) joints 2–5, and metatarsophalangeal (MTP) joints 1–5. To grade synovitis, we used the semi-quantitative scoring system introduced by Szkudlarek et al. (20) in which gray-scale synovial SH and hyperemia (PD) were graded on a scale of 0–3. We used the commonly applied definition of ultrasound arthritis of SH ≥2 and/or PD grade ≥1 as the cutoff for a pathologic ultrasound finding (8, 16, 21–24). PD signals were assessed only in joints with SH ≥1. Sum scores from the 36 investigated joints were calculated for SH and PD, respectively, resulting in a maximum score of 108 for both SH and PD.

In addition, three of the most commonly involved tendons in RA were examined bilaterally (extensor carpi ulnaris (ECU), tibialis posterior tendon (TPT), and common flexor digitorum longus (CFDL) in the feet) (25). Tenosynovitis was scored by GS according to OMERACT (8), and PD signals were scored as: 0 = none; 1 = minor; 2 = moderate; and 3 = major presence (25).

Regarding erosions, easily assessable and typical sites (MCP 2 and 5, ulnar head, PIP 2–5, and MTP 1 and 5) were dynamically examined on dorsal and lateral aspects, and erosions were reported as present (≥1) or not present. Erosion was defined as an interruption of the bone surface observed in two perpendicular planes with a diameter of ≥1 mm (8).

MTP I can be affected by concomitant conditions such as osteoarthritis. However, we chosen to report changes in this joint, since it is possible to distinguish typical erosive changes that do not rise above the bone surface from those degenerative changes with bone-proliferative features.

The ultrasound investigator did not participate in the clinical management of the patients, and the ultrasound results were blinded to both the patients and their respective physicians during the first 3 years of the study. Thereafter, they were available upon request. To determine the intra-reader reliability, baseline ultrasound images of 36 joints from 10 randomly chosen patients (in total 360 joints) were saved and re-assessed at least 2 weeks later, resulting in a kappa value of 0.948 for the presence of ultrasound synovitis (categorically as defined above), and 1.0 for the presence of erosions.

### Laboratory Analyses

Erythrocyte sedimentation rate (ESR) and C-reactive protein (CRP) were analyzed according to clinical routine practice at the Clinical Chemistry Laboratory, Linköping University Hospital. Agglutinating RF was analyzed by nephelometry at the accredited Clinical Immunology Laboratory, Linköping University Hospital (cutoff, 30 U/ml). In serum samples collected at baseline and stored at −80°C, anti-CCP antibodies were analyzed using the 2nd generation enzyme immunoassay (Immunoscan CCPlus; EuroDiagnostica AB, Malmö, Sweden). The cutoff was set at 25 AU/ml according to the manufacturer.

### Statistical Analyses

Statistical analyses were performed using the IBM SPSS Statistics 23 software. Continuous data were summarized with mean values and standard deviation, and non-normally distributed data with median values and IQR. Differences between groups were tested using the Student's *t*-test regarding continuous variables, and proportions were compared using the Chi-squared test. To assess the prognostic value of ultrasound features for clinical arthritis development, we performed univariable Cox regression analysis. Significant findings were further tested in a multivariable analysis that included baseline variables of potential importance for arthritis development (age, sex, symptom duration, RF status, ACPA levels, smoking habits, ESR, and CRP levels). Positive predictive values (PPV) and negative predictive values (NPV) for the ultrasound findings were calculated for significant associations in the multivariable model. Statistical significance was adjudged for two-sided *p*-values <0.05.

## Results

### Ultrasound Findings in Patients and Controls

At the joint level, significantly more MCP and PIP joints had SH ≥ 2 among the patients, as compared to the controls (Table 2). In contrast, SH ≥ 2 was more prevalent in MTP 1–5 among controls than among patients (30.2 vs. 18.7%, *p* < 0.001). Among the controls, SH was more frequent in MTP 1–4 than in any other location and was significantly over-represented compared to the patients (Table 2). Therefore, we decided to present MTP 1–4 separately from MTP 5, and to exclude MTP 1–4 from the analyses of SH vs. arthritis development in the patients.

**Table 2:** Comparison of ultrasound abnormalities at among anti-citrullinated protein antibody-positive at-risk patients vs. controls.

	**Synovial hypertrophy** **≥2**			**Power doppler** **≥1**		
**Joint(s)**	**Patients (*n* = 82)**	**Controls (*n* = 100)**	***p*-value**	**Patients (*n* = 82)**	**Controls (*n* = 100)**	***p*-value**
Wrist	8.9% (44/492)	7.0% (42/600)	0.259	7.9% (39/492)	2% (12/600)	**<0.001**
MCP 1-5	3.5% (29/820)	0.5% (5/1000)	**<0.001**	0.7% (6/820)	0% (0/1000)	**0.008**
PIP 2-5	5.0% (33/656)	0.6% (5/800)	**<0.001**	1.7% (11/656)	0% (0/800)	**<0.001**
MTP 1–4	22.1% (145/656)	37.4% (299/800)	**<0.001**	2.1% (14/656)	0.3% (2/800)	**0.001**
MTP 5	4.9% (8/164)	1.5% (3/200)	0.071	1.2% (2/164)	0% (0/200)	0.202
Total	9.3% (259/2788)	10.4% (354/3400)	0.146	2.6% (72/2788)	0.4% (14/3400)	**<0.001**
Total (excl. MTP 1–4)	5.3% 114/2132)	2.1% (55/2600)	**<0.001**	2.7% (58/2132)	0.5% (12/2600)	**<0.001**
Tendons	2.2% (11/492)	0.5% (3/600)	**0.014**	1.2% (6/492)	0% (0/600)	**0.008**

*MCP, Metacarpophalangeal joint; MTP, metatarsophalangeal joint; PIP, proximal interphalangeal joint of the finger. Significant p-values are shown in bold*.

PD signals (PD ≥ 1) were most commonly seen in wrists, i.e., radiocarpal, intercarpal, and/or radioulnar joints (7.9% of patient joints vs. 2.0% of control joints, *p* < 0.001), and were infrequent in other locations (≤ 3%; Table 2). A detectable PD signal (PD ≥ 1) at any location occurred in 37/82 (45%) of the patients, as compared to 5/100 (5%) of the controls (*p* < 0.001).

Tenosynovitis at baseline was found in 10/82 patients (ECU in 3 patients, TPT in 5, CFDL in 1, and both ECU and TPT in 1) and in 3/100 controls (ECU in 2, and CFDL in 1) (*p* = 0.021).

Ultrasound detected erosions in 13 patients (10 patients had 1, while 3 patients had 2 erosions), whereas, none of the controls had any erosions (*p* < 0.001, Table 3). Of the 16 erosions, 1 was localized in a PIP 2 joint radially, 4 in MCP 2 joints radially, 1 in MCP 5 joints ulnar, 4 in the head of ulna, 2 in MTP 1 medially, and 4 in MTP 5 joints laterally. At baseline, 6 of the 16 joints with bone erosions (38%) had synovitis according to ultrasound (SH ≥ 2 and/or PD ≥ 1). Conventional radiographs from baseline detected 1 out of the 16 (6%) bone erosions detected by ultrasound.

**Table 3:** Baseline ultrasound findings in patients without clinical arthritis at baseline compared to controls.

		**Patients (*N* = 82)**	**Controls (*N* = 100)**	***p*-value**
Hands	SH	4.0 (4.7)	2.1 (2.5)	**0.001**
	PD	1.0 (1.8)	0.04 (0.2)	**<0.001**
MTP1-4	SH	4.1 (4.1)	7.7 (4.3)	**<0.001**
	PD	0.2 (0.7)	0.04 (0.3)	**0.016**
MTP5	SH	0.3 (0.8)	0.2 (0.6)	0.462
	PD	0.04 (0.2)	0	0.181
Total (Hands + MTP1-5)	SH	8.4 (7.2)	9.9 (5.6)	0.102
Total (Hands + MTP1-5)	PD	1.3 (2.0)	0.1 (0.4)	**<0.001**
Tendons	SH	0.5 (1.1)	0.1 (0.4)	**0.005**
	PD	0.1 (0.6)	0 (0)	0.063
≥1 erosion present		13/82 (16%)	0 (0%)	**<0.001**

*Values shown are mean (standard deviation) sum scores unless otherwise indicated. Hands include wrists, metacarpophalangeal joints 1–5, and proximal interphalangeal joints 2–5. SH, Synovial hypertrophy; MTP, metatarsophalangeal joint; PD, power Doppler. Significant p-values are shown in bold*.

Table 3 summarizes the ultrasound findings at the patient level. The PD sum scores were higher in patients than in controls. SH showed site-specific differences: in the hands, the SH sum scores were higher among the patients, whereas, the SH sum scores in MTP 1–4 were higher among the controls. When excluding the feet, ultrasound-detected synovitis (defined as either SH ≥ 2 and/or PD ≥ 1) was noted in 55 patients (67%) and 33 controls (33%) (*p* < 0.001).

### Ultrasound Findings and Subsequent Arthritis Development

**Figure 2:**
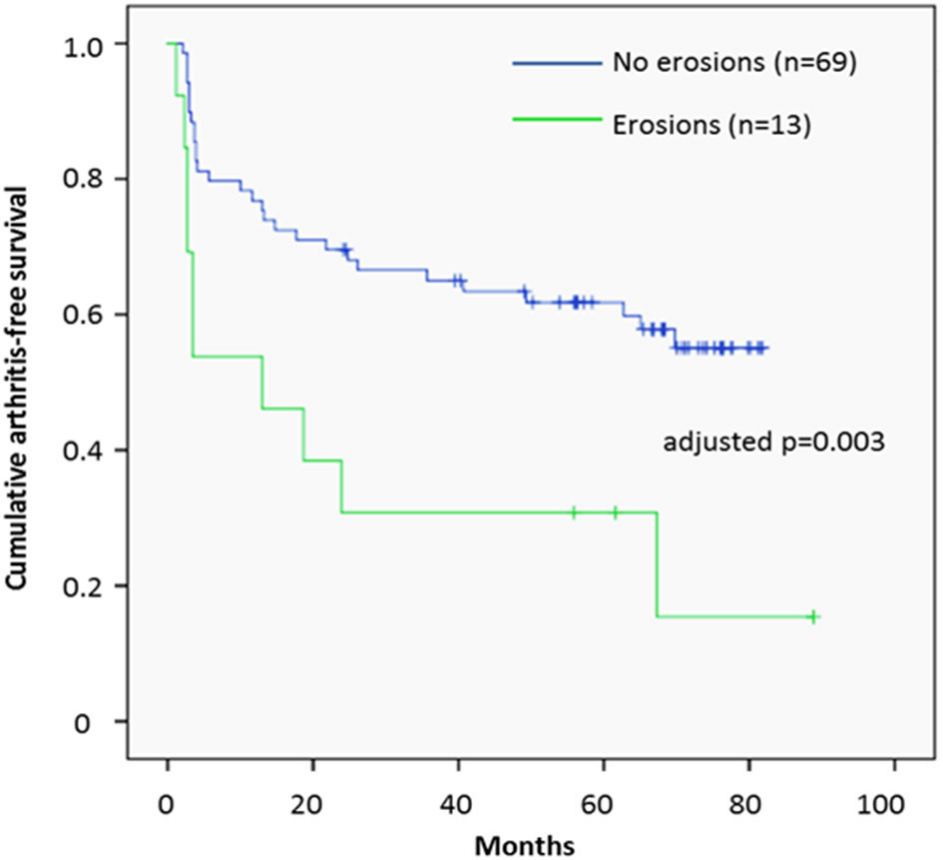
Clinical arthritis development in relation to baseline ultrasound erosions. Survival plot illustrating the development of clinical arthritis during follow-up in relation to the presence of ultrasound erosions at baseline among patients who had anti-citrullinated protein antibodies and musculoskeletal pain.

Ultrasound synovitis occurred in 55 patients (67%) when excluding the feet, and in 66 patients (81%) when including the feet. Neither the presence of ultrasound synovitis nor the SH or PD sum scores were significantly associated with the development of clinical arthritis (Table 4). However, 10 out of the 13 patients (77%) with ≥1 baseline erosion on ultrasound developed clinical arthritis during the follow-up period, as compared to 29/69 (42%) of those without erosions (*p* = 0.032). In the univariable Cox regression analysis, baseline erosions were associated with clinical arthritis development [Hazard Ratio (HR) 2.8, 95% CI 1.4–5.8, *p* = 0.005] (Table 4). We also tested whether erosions by ultrasound combined with inflammatory changes in joints and tendons increased the prognostic value concerning clinical arthritis development. Neither the HR for synovitis nor tenosynovitis in combination with bone erosions were higher than the HR for erosions alone (Table 4). After including potential confounders (sex, age, symptom duration, smoking habits, ESR, CRP levels, RF status, and ACPA levels) in the Cox regression model, the association between ultrasound-detected erosions and arthritis development remained statistically significant (HR 3.9, 95% CI 1.6–9.4, *p* = 0.003) (Figure 2). Since this model included a large number of variables (*n* = 10) in relation to events (*n* = 39), we also tested the prognostic value of erosions in a more strict multivariable model including CRP levels, RF status, and ACPA levels. Results remained very similar (Supplementary Table 1). The PPV for the development of arthritis in patients with baseline erosions was 77% and the NPV was 58%.

**Table 4:** Univariable Cox regression analysis of ultrasound findings with development of clinical arthritis as outcome.

**Ultrasound finding**	**Score/presence**	***N***	**Hazard ratio**	**95% CI**	***p*-value**
Synovial hypertrophy sum score	0–1	28	Reference		
	2–3	18	1.53	0.65–3.60	0.33
	≥4	36	1.48	0.70–3.13	0.31
Power Doppler sum score	0	45	Reference		
	≥1	37	1.68	0.89–3.15	0.11
Ultrasound synovitis	No	27	Reference		
	Yes	55	1.70	0.83–3.50	0.15
Ultrasound tenosynovitis	No	72	Reference		
	Yes	10	2.48	1.09–5.66	**0.031**
Ultrasound erosions	0	69	Reference		
	≥1	13	2.82	1.37–5.82	**0.005**
Erosions + synovitis	No	70	Reference		
	Yes	12	2.69	1.27–5.68	**0.010**
Erosion + tenosynovitis	No	79	Reference		
	Yes	3	2.76	0.66–11.6	0.165
Synovitis + tenosynovitis	No	73	Reference		
	Yes	9	2.23	0.93–5.36	0.072
Erosion + synovitis + tenosynovitis	No	79	Reference		
	Yes	3	2.76	0.66–11.6	0.165

*Ultrasound synovitis and ultrasound tenosynovitis are defined as SH ≥ 2 and/or PD ≥ 1. Significant p-values are shown in bold*.

Seven patients started treatment with DMARDs or oral corticosteroids during the follow-up despite no confirmed arthritis upon clinical examination. When we performed a multivariable Cox regression analyses while excluding these patients, erosions remained significantly associated with arthritis development (HR 4.2; 95% CI 1.7–10.0, *p* = 0.001), while SH and PD were still not significantly associated with arthritis development. In another sensitivity analysis, we restricted the analysis to the initial 3 years when the ultrasound results were completely blinded. During this period, 32/82 patients (39%) developed clinical arthritis, and the association with baseline ultrasound-detected erosions remained significant in the multivariable analysis (HR 3.5, 95% CI 1.3–9.0, *p* = 0.011).

The presence of baseline tenosynovitis in patients was associated with the development of clinical arthritis in the univariable analysis (Table 4). However, it did not remain statistically significant in the multivariable analysis (HR 1.93, 95% CI 0.75–4.97, *p* = 0.18).

## Discussion

This prospective observational study identifies ultrasound-detected bone erosions as an independent prognostic factor for clinical arthritis development in ACPA-positive at-risk patients without signs of clinical arthritis at baseline. This association persisted when other known predictors were considered, suggesting that ultrasound scanning for erosions is a valuable tool to risk-stratify ACPA-positive patients with MSK pain, at least concerning the outcome of clinical arthritis. Whether or not ultrasound erosions also predict progression of structural joint damage should be addressed in future studies.

Gray-scale ultrasound findings of SH were not significantly associated with progression to clinical arthritis in the current study, and the existing literature concerning the prognostic value of SH is divergent. Van der Stadt et al. (12) did not find a predictive value for GS at the patient level, while two studies have reported significant associations with arthritis development, albeit only after excluding the feet (14, 26). A recent Dutch study has shown a predictive value for SH in combination with PD, although SH was not reported separately (22). From the healthy controls included in the current study, we conclude that SH is a common finding, also when looking outside the feet (27). Therefore, findings of SH must be interpreted with caution and not *per se* be regarded as “ultrasound synovitis.”

While over-represented among the patients, the PD findings also failed to show a significant prognostic value. As for SH, the literature regarding PD includes both studies that demonstrate significant associations with arthritis development (22) and those that do not (12, 14), although all report numerically increased risk estimates. Differences in ultrasound equipment may influence PD performance across studies, and more recently introduced devices may have superior PD sensitivity than the device used in our study. Nevertheless, we conclude that PD is more specific than SH when comparing ACPA-positive MSK patients to similarly aged controls without MSK pain, but larger studies are warranted to characterize more precisely the possible prognostic value of PD signals in at-risk patients.

Recent data suggests that inflammatory tendon abnormalities are uncommon in healthy subjects (28), which is in line with our findings among healthy controls. Previous data on tenosynovitis in at-risk patients are scarce. We found an increased prevalence among ACPA-positive patients, and a significant association with progression to clinical arthritis. However, the multivariable analysis did not confirm an independent prognostic value.

Ultrasound erosions were very specific findings in our study, being detected in 16% of the ACPA-positive patients with MSK but not in any of the controls. Our results are in line with the study of Nam et al., in which none of the 48 controls had erosions in any of the examined joints in the hands or wrist, only two had a small erosion in one of their fifth MTP joints (14). Since bone-specific effects of ACPA have been discussed extensively in recent years (29, 30), it is intriguing that the ultrasound feature with the strongest prognostic value in our ACPA-positive study population reflects bone damage rather than inflammation. Fewer than half of the joints with ultrasound-detected erosions concurrently had ultrasound-detected synovitis (and none had clinical arthritis), which is compatible with the hypothesis of structural damage preceding arthritis in at least a subset of ACPA-positive individuals (31). The one previous study on ultrasound erosions in ACPA-positive at-risk patients found a HR very similar to ours (14). Taken together, the studies strongly support the use of ultrasound scanning for erosions in ACPA-positive patients with MSK symptoms to improve prognostic capability. Given the general benefit of early initiation of anti-rheumatic therapy in patients with RA, the issue as to whether patients with ultrasound erosions would benefit from very early pharmacotherapy needs to be addressed in future studies.

A strength of the current study is the long follow-up period, which increases the chances to identify those at risk of developing arthritis after several years. In addition, the large control population places the ultrasound data in perspective, for instance by demonstrating that SH in MTP 1–4 must be interpreted with caution. Furthermore, the ultrasound results were blinded to the patients and the treating physicians, thereby removing the risk of influencing clinical judgement and treatment decisions.

A limitation of the study is that treatment was not defined in the study protocol. Importantly, however, the analyses that excluded the seven patients who were subjected to corticosteroid and/or DMARD therapy without a confirmed clinical arthritis did not alter the results in any substantial way. A second limitation of this work is the relatively small sample size, resulting in rather wide CIs and difficulties to reliably look into subgroups of patients. Another potential limitation is the fact that there was only one ultrasound investigator, who was not blinded to participant status (patient vs. control). However, the ultrasound sonographer still graded controls with more SH in the MTP joints, and we therefore not believe that non-blinding resulted in overrated findings among patients compared to controls. Finally, due to practical reasons, arthritis development was not confirmed by a second investigator or compared with ultrasound findings in the same joint. However, the clinical investigators were experienced, and patients were seen by the same doctor at most of the visits.

## Conclusions

We conclude that bone erosions detected by ultrasound are independent predictors for the development of clinical arthritis in ACPA-positive patients with MSK pain and without baseline arthritis. Thus, ultrasound examinations in this clinical setting should include assessments of bone erosions, in order to improve risk stratification.

## Data Availability Statement

The raw data supporting the conclusions of this article will be made available by the authors upon reasonable request.

## Ethics Statement

The studies involving human participants were reviewed and approved by Regional Ethics Committee in Linköping, Sweden (DnR 220-09 and 2015/236-32). The patients/participants provided their written informed consent to participate in this study.

## Author Contributions

MZ, TS, and AK conceived the study. MZ performed the ultrasound examinations and was responsible for study coordination. EE, JC, ÅR, TS, and AK were involved in the recruitment and characterization of patients. HH and MZ developed the ultrasound protocols. MM and MZ performed statistical analyses. KM was responsible for the laboratory analyses. MZ, MM, and AK drafted the manuscript and received critical input from all the co-authors. All authors contributed to the article and approved the submitted version.

## Conflict of Interest

The authors declare that the research was conducted in the absence of any commercial or financial relationships that could be construed as a potential conflict of interest.
