# Supplementary material for: Bone Erosions Detected by Ultrasound Are Prognostic for Clinical Arthritis Development in Patients With ACPA and Musculoskeletal Pain
Source: Front Med (Lausanne). 2021 Mar 23;8:653994. doi: 10.3389/fmed.2021.653994 (PMC8021704; doi:10.3389/fmed.2021.653994)
Supplement: Supplementary file 1 [file Table_1.DOCX]

**Suppl Table 1** Cox regression analysis of factors predicting development of clinical arthritis.

| ***Variable*** | ***Simple Cox regression*** | | | ***Multiple Cox regression*** | | |
| --- | --- | --- | --- | --- | --- | --- |
|  | ***95% CI*** | ***HR*** | ***P value*** | ***95% CI*** | ***HR*** | ***P value*** |
| Erosions by US | 1.4-5.8 | 2.8 | 0.005 | 1.4-6.3 | 3.0 | 0.005 |
| Age | 1.0-1.0 | 1.0 | n.s. | 1.0-1.0 | 1.0 | n.s. |
| ACPA levels | 1.0-1.0 | 1.0 | <0.001 | 1.0-1.0 | 1.0 | 0.006 |
| RF positivity | 1.2-4.4 | 2.3 | 0.010 | 1.0-1.0 | 1.0 | <0.001 |
| CRP | 1.0-1.1 | 1.1 | 0.001 | 0.9-1.0 | 1.0 | n.s. |
| ACPA, Anti-citrullinated protein antibodies; CRP, C-reactive protein; HR, Hazard ratio; RF, rheumatoid factor; US, ultrasound. | | | | | | |
